# Vogel-Fulcher-Tamman criticality of 3D superinsulators

**DOI:** 10.1038/s41598-018-33765-5

**Published:** 2018-10-24

**Authors:** M. C. Diamantini, L. Gammaitoni, C. A. Trugenberger, V. M. Vinokur

**Affiliations:** 1 NiPS Laboratory, INFN and Dipartimento di Fisica e Geologia, University of Perugia, via A. Pascoli, I-06100 Perugia, Italy; 2 SwissScientific Technologies SA, rue du Rhone 59, CH-1204 Geneva, Switzerland; 3 Materials Science Division, Argonne National Laboratory, 9700 S. Cass Ave, Argonne, IL 60439 USA

## Abstract

It has been believed that the superinsulating state, which is the low-temperature charge Berezinskii-Kosterlitz-Thouless (BKT) phase, can exist only in two dimensions. We develop a general gauge description of the superinsulating state and the related deconfinement transition of Cooper pairs and predict the existence of the superinsulating state in three dimensions (3d). We find that 3d superinsulators exhibit Vogel-Fulcher-Tammann (VFT) critical behavior at the phase transition. This is the 3d string analog of the Berezinski-Kosterlitz-Thouless (BKT) criticality for logarithmically and linearly interacting point particles in 2d. Our results show that singular exponential scaling behaviors of the BKT type are generic for phase transitions associated with the condensation of topological excitations.

## Introduction

The superinsulating state, having infinite resistance at finite temperatures^1–5^ is the state dual to superconductivity, endowed with a finite temperature infinite conductance. Originally^1,2^, the emergence of superinsulation was attributed to logarithmic Coulomb interactions in two spatial dimensions (2d) arising from the dimensional reduction of the effective Coulomb interactions due to the divergence of the dielectric constant *ε*^3,4^ near the superconductor-insulator transition (SIT)^6–12^ in disordered superconducting films. A more recent approach^13^ based on the condensation of magnetic monopoles^14^, however, derived superinsulation as a result of the linear confinement of Cooper pairs by electric strings^15,16^, which are the S-dual of Abrikosov vortices in superconductors^15,17,18^. This offers a more general view of superinsulation as a phenomenon that is not specific to two dimensions but can exist also in 3d systems and confirms the original idea of ’t Hooft of superinsulation as the prototype of confining mechanisms via dual superconductivity^19^.

This immediately poses a question about the experimental effect that could serve as a hallmark of superinsulation and that could, at the same time, unequivocally discriminate between the 3d and 2d superinsulators, exposing the linear nature of the underlying confinement. In disordered superconducting films that host superinsulating state at the insulating side of the superconductor-insulator transition (SIT), it is the charge Berezinskii-Kosterlitz-Thouless (BKT) transition^20–23^ that marks the emergence of the superinsulating state^3,4^ and which was detected experimentally^5^ by the BKT critical behavior^24^ of the conductance $G\propto \exp [-\,b/\sqrt{|T/{T}_{{\rm{CBKT}}}-\mathrm{1|}}]$, where *T*_CBKT_ is the temperature of the charge BKT transition and *b* is a constant of order unity. This suggests that it is the conductance critical behavior that provides the criterion for identifying the superinsulating state.

The BKT critical scaling of the conductance follows from the exponential critical scaling of the correlation length^24^

1 $${\xi }_{\pm }\propto \exp [\frac{{b}_{\pm }}{\sqrt{|T/{T}_{{\rm{c}}}-\mathrm{1|}}}],$$

where ± subscript labels *T* > *T*_c_ and *T* < *T*_c_ regions respectively, and *ξ*_−_ is interpreted as the maximum size of the bound charge-anticharge pair. It is known^25^ that, in two dimensions, both logarithmic and linear confinement lead to the BKT critical scaling, so we use the notation *T*_c_ for either the BKT or deconfinement transition temperature. Our goal is now to reveal how the deconfinement scaling of Eq. (1) evolves in 3d systems. We show below that in 3d the critical behaviour of superinsulators is modified to the Vogel-Fulcher-Tammann (VFT) critical form^26^

2 $${\xi }_{\pm }\propto \exp (\frac{{b^{\prime} }_{\pm }}{|T/{T}_{{\rm{c}}}-\mathrm{1|}}).$$

This behaviour is characteristic of the one-dimensional confining strings in 3d, where the world-surface elements interact logarithmically as particles in 2d. The VFT scaling is typical of glassy systems and has recently been derived^27^ for the 3d XY model with quenched disorder. Here we show that it arises naturally, without assuming any disorder, for confining strings and is thus a signature for 3d superinsulators.

## Confining Strings

The electromagnetic effective action of a superinsulator is given by^13^ ${S}^{SI}\propto {\sum }_{x,\mu ,\nu }\,[1-\,\cos \,\mathrm{(2}e{\ell }^{2}{F}_{\mu \nu })]$, where {*x*} represent the sites a *d*-dimensional lattice, *ℓ* is the corresponding lattice spacing, *e* is the electron charge, and *F*_*μν*_ is the electromagnetic field strength. This is Polyakov’s compact QED action^15,16^: hence the conclusion^13^ that, in superinsulators, Cooper pair dipoles are bound together into neutral “mesons” by Polyakov’s confining strings^28^. These strings have an action which is induced by coupling their world-sheet elements to a massive Kalb-Ramond tensor gauge field^29^. They can be explicitly derived for compact QED^30^, the induced electromagnetic action for the superinsulator^13^ and for Abelian-projected SU(2)^31,32^. Their world-sheet formulation is thus in term of a non-local, long-range interaction between surface elements^33^. Best suited to derive physical and geometric properties of these strings, however is the corresponding derivative expansion truncated to a certain level *n*^34^ (we use natural units *c* = 1, ℏ = 1),

3 $$\begin{array}{rcl}S & = & \int \,{d}^{2}\xi \sqrt{g}{g}^{ab}{{\mathscr{D}}}_{a}{x}_{\mu }{V}_{n}({{\mathscr{D}}}^{2}){{\mathscr{D}}}_{b}{x}_{\mu },\\ {V}_{n}({{\mathscr{D}}}^{2}) & = & t{{\rm{\Lambda }}}^{2}+\sum _{k=1}^{2n}\,\frac{{c}_{k}}{{{\rm{\Lambda }}}^{2k-2}}{({{\mathscr{D}}}^{2})}^{k}.\end{array}$$

here *g*_*ab*_ = ∂_*a*_*x*_*μ*_∂_*b*_*x*_*μ*_ is the induced metric of the world-sheet **x**(*ξ*_0_, *ξ*_1_) in *D* = *d* + 1 Euclidean dimensions, *g* the corresponding determinant and ${{\mathscr{D}}}_{a}$ the corresponding covariant derivatives. *V*_*n*_(${{\mathscr{D}}}^{2}$) represents the truncation of the non-local interaction at level *n* and *λ* is the ultraviolet (UV) cutoff. The quantity 2*t* is the bare surface tension. Since *c*_*k*_ are alternating in sign^33^, energy stability considerations dictate that the truncation must terminate with an even *k* = 2*n*. The second coefficient is the stiffness, representing the string rigidity. In confining strings it is actually *negative*. The string is stabilized by the last term in the truncation which generates a string tension ∝ Λ^2^/*c*_2*n*_ which dominates fluctuations at large scales and leads to long range correlations, thus avoiding the crumpling affecting most string models^35^. In the simplest version with *n* = 1, the third term in the derivative expansion is the string hyperfine structure which contains (${{\mathscr{D}}}^{2}$) and, therefore, suppresses spikes, since the latter imply huge gradients of the extrinsic curvature.

The only restriction imposed on the free coefficients *c*_*k*_ is the absence of both tachyons and ghosts, which can be fulfilled by requiring that the Fourier transform *V*_*n*_(p^2^) has no zeros on the real p^2^-axis. As a consequence, the polynomial *V*_*n*_(p^2^) must then have *n* pairs of complex-conjugate zeros in the complex p^2^-plane. The associated mass scales represent the *n* string resonances determining the string structure on finer and finer scales, the first resonance being the hyperfine structure. Increasing *n* amounts thus to measuring the string on ever finer scales. To make computations more transparent we will set all coefficients with odd *k* to zero, *c*_2*m*+1_ = 0 for 0 ≤ *m* ≤ *n* − 1, which is anyway their value at the infrared-stable fixed point^35^. Of course, when deriving the confining string from compact QED, all coefficients *c*_*k*_ are fixed in terms of the only two dimensionless parameters available, *e*/Λ^2−*D*/2^, with *e* the QED coupling constant, and the monopole fugacity z^2^. In particular^33^

4 $$t=\frac{{z}^{2}}{\mathrm{4(2}\pi {)}^{D\mathrm{/2}-1}}{\tau }^{D\mathrm{/2}-2}{K}_{D\mathrm{/2}-2}(\tau ),$$

where *τ* = Λ^2−*D*/2^*z*/*e*.

In the following we will consider the confining string model at finite temperatures in the large *D* approximation. High temperatures were considered in^34^, where it was shown that the corresponding behaviour matches the expected high-temperature behaviour of large *N* QCD^36^. Here we will, instead, concentrate on the critical behaviour at the deconfinement transition, where the renormalised string tension vanishes and strings become infinitely long on the cutoff scale.

## Finite Temperature Behaviour

In the following we will consider the fluctuation contribution *S*_1_ to the large-*D* string action derived in Methods for temperatures such that

5 $$\frac{{c}_{2n}}{{{\rm{\Lambda }}}^{4n-2}}\frac{1}{{\beta }^{4n}}\gg {{\rm{\Lambda }}}^{2}t+\sum _{k=1}^{2n-1}\,\frac{{c}_{k}}{{{\rm{\Lambda }}}^{2k-2}}\frac{1}{{\beta }^{2k}}.$$

For *l* ≠ 0, the fluctuation term *S*_1_ is dominated by the highest-order term in the derivative expansion. This can be obtained by using analytic regularization and analytic continuation of the expression ${\sum }_{n=1}^{\infty }\,{n}^{-z}=\zeta (z)$ for the Riemann zeta function, with *ζ*(−1) = −1/12,

6 $$\begin{array}{ll} & \frac{D-2}{2}R\sqrt{{\rho }_{1}}\,\sum _{l=-\infty }^{+\infty }\,\int \,\frac{d{p}_{1}}{2\pi }\,\mathrm{ln}\,\frac{{c}_{2n}}{{{\rm{\Lambda }}}^{4n-2}}{({\omega }_{l}^{2}+{p}_{1}^{2})}^{2n+1}\\  & =\,\frac{D-2}{2}\sqrt{\frac{{\rho }_{1}}{{\rho }_{0}}}\mathrm{(2}n+\mathrm{1)4}\pi \frac{R}{\beta }\sum _{l=1}^{+\infty }\,\sqrt{{l}^{2}}\end{array}$$

7 $$=\,\frac{-D-2}{2}\sqrt{\frac{{\rho }_{1}}{{\rho }_{0}}}\frac{\mathrm{(2}n+\mathrm{1)}\pi }{3}\frac{R}{\beta }.$$

For *l* = 0, the computation of the corresponding term

8 $${S}_{1}=\frac{D-2}{2}R\sqrt{{\rho }_{1}}\int \,\frac{d{p}_{1}}{2\pi }\,\mathrm{ln}({p}_{1}^{2}{\bar{V}}_{n}({p}_{1}^{2})),$$

with

9 $${\bar{V}}_{n}({p}_{1}^{2})=({{\rm{\Lambda }}}^{2}(t+{\lambda }_{1})+\sum _{k=1}^{2n}\,\frac{{c}_{k}}{{{\rm{\Lambda }}}^{2k-2}}{p}_{1}^{2k}),$$

requires a bit more care. Since *c*_*k*_ = 0 for all odd *k* and the model is subject to the requirement that the action must be ghost- and tachyon-free, all pairs of complex-conjugate zeros of ${\bar{V}}_{n}({p}_{1}^{2})$ lie on the imaginary axis and thus^35^ ${\bar{V}}_{n}({p}_{1}^{2})$ can be expressed as

10 $$\frac{{{\rm{\Lambda }}}^{4n-2}}{{c}_{2n}}\,{\bar{V}}_{n}({p}_{1}^{2})=\prod _{k=1}^{n}({p}_{1}^{4}+{\alpha }_{k}^{2}{{\rm{\Lambda }}}^{4}),$$

with purely numerical coefficients *α*_*k*_. Using again analytic regularization and the analytic continuation of the Riemann zeta function we obtain

11 $$\begin{array}{rcl}{S}_{1} & = & \frac{D-2}{2}R\sqrt{{\rho }_{1}}\sum _{k=1}^{n}\,\int \,\frac{d{p}_{1}}{2\pi }\,\mathrm{ln}({p}_{1}^{4}+{\alpha }_{k}^{2}{{\rm{\Lambda }}}^{4})\\  & = & \frac{D-2}{2}R\sqrt{{\rho }_{1}}\sum _{k=1}^{n}\,\int \,\frac{d{p}_{1}}{2\pi }2{\rm{Re}}\,\mathrm{ln}({p}_{1}^{4}+i{\alpha }_{k}{{\rm{\Lambda }}}^{2})\\  & = & \frac{D-2}{2}R\sqrt{{\rho }_{1}}\sum _{k=1}^{n}\,{\rm{\Lambda }}\sqrt{2{\alpha }_{k}}.\end{array}$$

At this point we can resum the fluctuation contribution *S*_1_ with the zeroth-order term (see Methods). Taking into account both the *l* = 0 and the *l* ≠ 0 contributions we obtain

12 $$S={S}_{0}+\frac{D-2}{2}R\sqrt{{\rho }_{1}}[\sum _{k=1}^{n}\,{\rm{\Lambda }}\sqrt{2{\alpha }_{k}}-\frac{\mathrm{(2}n+\mathrm{1)}\pi }{3\sqrt{{\rho }_{0}}}\frac{1}{\beta }].$$

The coefficients in the expression (10) are not completely free. In order to match (9), the *p*_1_-independent term has to be compatible with

13 $$\prod _{k=1}^{n}\,{\alpha }_{k}^{2}{{\rm{\Lambda }}}^{4}=\frac{{{\rm{\Lambda }}}^{4n}}{{c}_{2n}}(t+{\lambda }_{1}\mathrm{)}.$$

For simplicity’s sake we shall also make the assumption that all coefficients *α*_*k*_ are equal to each other, implying thereby that there is a unique resonance that determines the fine details of the string oscillations. Then (13) implies:

14 $${\alpha }_{k}^{2}={(t+{\lambda }_{1})}^{\mathrm{1/}n}{\alpha }^{2},\,\alpha ={(\frac{1}{{c}_{2n}})}^{\mathrm{1/2}n}.$$

Since the fluctuations contribution *S*_1_ is proportional to (*D* − 2), it is forced onto its ground state in the large *D* limit. In this limit the metric components *ρ*_0_ and *ρ*_1_ and the Lagrange multipliers *λ*_0_ and *λ*_1_ take on their classical values obtained by setting the respective derivatives of the total action to zero. This gives four large-*D* gap equations

15 $$\frac{1-{\rho }_{0}}{{\rho }_{0}}=0,$$

16 $$\frac{1}{{\rho }_{1}}=1-\frac{D-2}{2}\frac{1}{4\beta {\rm{\Lambda }}}\sqrt{2\alpha }{({\lambda }_{1}+t)}^{\mathrm{1/4}n-1},$$

17 $$[\frac{1}{2}(t-{\lambda }_{1})+\frac{1}{2{\rho }_{1}}({\lambda }_{1}+t)-t-{\lambda }_{0}]+\frac{D-2}{2}\frac{\mathrm{(2}n+\mathrm{1)}\pi }{6{\beta }^{2}{{\rm{\Lambda }}}^{2}}=0,$$

18 $$(t-{\lambda }_{1})-\frac{1}{{\rho }_{1}}({\lambda }_{1}+t)+\frac{D-2}{2}\frac{1}{\beta {\rm{\Lambda }}}[\sqrt{2\alpha }\,n{({\lambda }_{1}+t)}^{\mathrm{1/4}n}-\frac{\pi \mathrm{(2}n+\mathrm{1)}}{3\beta {\rm{\Lambda }}}]=0.$$

Inserting (18) and (15) into (12) and using *ρ*_0_ = 1 from (15) we obtain the action in the form

19 $$S={A}_{{\rm{ext}}}\,{\mathscr{T}},$$

with ${\mathscr{T}}={{\rm{\Lambda }}}^{2}\mathrm{2(}{\lambda }_{1}+t)/\sqrt{{\rho }_{1}}={{\mathscr{T}}}_{0}/\sqrt{{\rho }_{1}}$ representing the renormalized string tension, expressed in terms of the zero-temperature renormalized string tension *T*_0_. Equation (16) for the spatial metric can be reformulated in the limit *n* → ∞ as

20 $$\frac{1}{{\rho }_{1}}=1-\frac{D-2}{2}T\frac{\sqrt{2\alpha }{\rm{\Lambda }}}{4}\frac{1}{({\lambda }_{1}+t){{\rm{\Lambda }}}^{2}},$$

From here we recognize that the renormalization of the string requires taking the simultaneous limits (*λ*_1_ + *t*) → 0, $\sqrt{2\alpha }\to 0$ and Λ → ∞ so that (*λ*_1_ + *t*)Λ^2^ and $\sqrt{2\alpha }{\rm{\Lambda }}$ are finite. In this case both the *ρ*_1_ metric element and the renormalized string tension acquire finite values. The scale $\sqrt{2\alpha }{\rm{\Lambda }}$ represents the renormalized mass *M* of the string resonance that determines, together with *T*_0_ all physical properties of the string. In particular, the finite temperature deconfinement critical behaviour is obtained as the limit (*λ*_1_ + *t*) → 0. In this limit the strings become infinitely long on the scale of the cutoff and the particles at their ends are liberated. The critical behaviour is embodied by the behaviour of the (dimensionless) correlation length $\xi =\mathrm{1/}\sqrt{{\lambda }_{1}+t}$ near the critical temperature.

## Critical Behaviour

In order to study the critical behaviour we derive the gap equation for (*λ*_1_ + *t*) alone, by substituting (16) into (18). This gives

21 $$({\lambda }_{1}+t)-\frac{D-2}{2}\frac{4n+1}{8\beta {\rm{\Lambda }}}\sqrt{2\alpha }{({\lambda }_{1}+t)}^{\frac{1}{4n}}+\frac{D-2}{2}\frac{2n+1}{6}\frac{\pi }{{\beta }^{2}{{\rm{\Lambda }}}^{2}}-t=0.$$

For $({\lambda }_{1}+t)\ll 1$ the first term can be neglected with respect to the second for large $n\gg 1$, which gives

22 $$4n\sqrt{2\alpha }{({\lambda }_{1}+t)}^{\frac{1}{4n}}=n\frac{8\pi }{3}\frac{T}{{\rm{\Lambda }}}-\frac{2}{D-2}\frac{8t{\rm{\Lambda }}}{T}.$$

Dividing by $\sqrt{2\alpha }$ and subtracting on both sides of the equation a term 4*n* we obtain

23 $$4n[{({\lambda }_{1}+t)}^{\frac{1}{4n}}-1]=\frac{8\pi n}{3}\frac{T}{M}[1-\frac{3}{(D-\mathrm{2)}\pi }\frac{\vartheta }{{T}^{2}}-\frac{3}{2\pi }\frac{M}{T}],$$

where *ϑ* = 2*t*Λ^2^/*n* is the bare string tension divided by *n*. The expression in square brackets on the right hand side can be formulated as

24 $$[1-\frac{3}{(D-\mathrm{2)}\pi }\frac{\vartheta }{{T}^{2}}-\frac{3}{2\pi }\frac{M}{T}]=(1-\frac{{T}_{+}}{T})(1+\frac{{T}_{-}}{T}),$$

where

25 $${T}_{\pm }=\frac{4}{(D-\mathrm{2)}}\frac{\vartheta }{M}\frac{1}{\mp 1+\sqrt{1+\frac{16\pi }{\mathrm{3(}D-\mathrm{2)}}\frac{\vartheta }{{M}^{2}}}}.$$

From here we read off the critical deconfinement temperature as *T*_*c*_ = *T*_+_. As expected it is determined by a combination of the two mass scales $\sqrt{\vartheta }$ and *M* in the model. Expanding the left-hand side of (23) around this critical temperature we get

26 $$4n[{({\lambda }_{1}+t)}^{\frac{1}{4n}}-1]=\frac{8\pi }{3}[1+(\frac{{T}_{-}}{T})](\frac{-n{\rm{\Delta }}T}{M})-O(n{\rm{\Delta }}{T}^{2}),$$

where Δ*T* = *T*_*c*_ − *T*. Taking the limit *n* → ∞, one obtains on the left-hand side ln(*λ*_1_ + *t*). The right-hand side, however, requires more care. Clearly, we recognize immediately that increasing *n* → ∞ drives the string to its critical point Δ*T* = 0. To establish in detail how this occurs, however, we must resort to the behaviour (4) of the bare string tension. For the relevant regime of small *τ* (strong coupling and low monopole fugacity) the quantity *ϑ* appearing in the equations above is given by

27 $$\begin{array}{rcl}{\vartheta }_{D=3} & = & \frac{ze}{8}\frac{{{\rm{\Lambda }}}^{\frac{3}{2}}}{n},\\ {\vartheta }_{D=4} & = & \frac{{z}^{2}}{4\pi }\,\mathrm{ln}(\frac{2e}{z})\frac{{{\rm{\Lambda }}}^{2}}{n},\end{array}$$

The zero-temperature fixed point being given by $\ell =1/{\rm{\Lambda }}=0$, this show that *n* must scale as $n\propto {\ell }^{-\mathrm{3/2}}$ for *D* = 3 and $n\propto {\ell }^{-2}$ for *D* = 4 in the approach to a fixed point. Correspondingly, we have *n* ∝ (Δ*T*)^−3/2^ for *D* = 3 and *n* ∝ (Δ*T*)^−2^ for *D* = 4 when approaching the finite temperature critical point. Therefore the gap equation

28 $$\mathrm{ln}({\lambda }_{1}+t)=\frac{8\pi }{3}[1+(\frac{{T}_{-}}{T})]{\mathrm{lim}}_{n\to \infty }(\frac{-n{\rm{\Delta }}T}{M})+\ldots ,$$

leads directly to the critical scaling behaviours given by Eq. (1) and Eq. (2) when approaching the deconfinement transition from below, reproducing thus the BKT criticality predicted in *d* = 2 by the Svetitsky-Yaffe conjecture^25^ and the VFT criticality of the deconfinement transition in *d* = 3.

Remarkably, exactly this 3d-like signature has been recently observed^37^ for the finite temperature insulating phase in InO disordered films, in which the thickness is much larger than the superconducting coherence length. While it seems premature to view this result as a conclusive evidence, yet one can view it as a possible indication of linear confinement in 3d superinsulators. Another important corollary of our results is that the disorder strength in disordered superconducting films plays the role of a parameter tuning the strength of the Coulomb interactions but that disorder in itself is irrelevant for the nature of the various phases around the SIT. Finally, an important and deep implication of our findings is that, since the VFT behavior of Eq. (2) is recognized as heralding glassy behavior, our results suggest that, in 3d, topological defects endowed with long range interactions generate a glassy state without any quenched disorder. Note that the VFT behavior in 3d superinsulators arises as characteristic of the deconfinement transition of a strongly interacting gauge theory. The putative glassy behavior below this transition heralds the formation of a *quantum glass* arising due to the condensation and entanglement of extended string-like topological excitations.

## Methods

### The large D approximation

In this Methods section we refresh the standard large-*D* approximation technique for strings. Following^38,39^, we introduce a Lagrange multiplier *λ*^*ab*^ to enforce the constraint that the induced metric ∂_*a*_*x*_*μ*_∂_*b*_*x*_*μ*_ be equal to the intrinsic metric *g*_*ab*_,

29 $$S\to S+\int {d}^{2}\xi \sqrt{g}[{{\rm{\Lambda }}}^{2}{\lambda }^{ab}({\partial }_{a}{x}_{\mu }{\partial }_{b}{x}_{\mu }-{g}_{ab})].$$

We then parametrize the world-sheet in a Gauss map by *x*_*μ*_(*ξ*) = (*ξ*_0_, *ξ*_1_, *ϕ*^*i*^(*ξ*)), *i* = 2, ..., *D* − 2. *ξ*_0_ is a periodic coordinate satisfying −*β*/2 ≤ *ξ*_0_ ≤ *β*/2, with *β* = 1/*T*, where *T* is the temperature. The space coordinate satisfies −*R*/2 ≤ *ξ*_1_ ≤ *R*/2, where *R* is the string length. The remaining coordinates *ϕ*^*i*^(*ξ*) describe the *D* − 2 transverse string fluctuations.

We now search for a saddle-point solution with diagonal metric *g*_*ab*_ = diag (*ρ*_0_, *ρ*_1_), and a Lagrange multiplier taking the form *λ*^*ab*^ = diag (*λ*_0_/*ρ*_0_, *λ*_1_/*ρ*_1_). With this Ansatz, the action simplifies to a sum of a tree-level contribution *S*_0_ and a fluctuations contribution *S*_1_,

30 $$\begin{array}{rcl}S & = & {S}_{0}+{S}_{1}\\ {S}_{0} & = & {A}_{{\rm{e}}xt}\,{{\rm{\Lambda }}}^{2}\sqrt{{\rho }_{0}{\rho }_{1}}[t(\frac{{\rho }_{0}+{\rho }_{1}}{{\rho }_{0}{\rho }_{1}})+{\lambda }_{0}(\frac{1-{\rho }_{0}}{{\rho }_{0}})+{\lambda }_{1}(\frac{1-{\rho }_{1}}{{\rho }_{1}})],\\ {S}_{1} & = & \int {d}^{2}\xi \sqrt{g}[{g}^{ab}{\partial }_{a}{\varphi }^{i}{V}_{n}({{\mathscr{D}}}^{2}){\partial }_{b}{\varphi }^{i}+{{\rm{\Lambda }}}^{2}{\lambda }^{ab}{\partial }_{a}{\varphi }^{i}{\partial }_{b}{\varphi }^{i}],\end{array}$$

with *βR* = *A*_e*xt*_ the extrinsic area in coordinate space. Integrating over the transverse fluctuations in the limit *R* → ∞ we get

31 $${S}_{1}=\frac{D-2}{2}R\sqrt{{\rho }_{1}}\sum _{l=-\infty }^{+\infty }\int \frac{d{p}_{1}}{2\pi }\,\mathrm{ln}[({p}_{1}^{2}{\lambda }_{1}+{\omega }_{l}^{2}{\lambda }_{0}){{\rm{\Lambda }}}^{2}+{p}^{2}{V}_{n}({p}^{2})]\,,$$

where $${p}^{2}={p}_{1}^{2}+{\omega }_{l}^{2}$$, and $${\omega }_{l}=2\pi \beta \sqrt{{\rho }_{0}}l$$.
